# Overexpressed PRAME is a potential immunotherapy target in sarcoma subtypes

**DOI:** 10.1186/s13569-017-0077-3

**Published:** 2017-06-15

**Authors:** Jason Roszik, Wei-Lien Wang, John A. Livingston, Christina L. Roland, Vinod Ravi, Cassian Yee, Patrick Hwu, Andrew Futreal, Alexander J. Lazar, Shreyaskumar R. Patel, Anthony P. Conley

**Affiliations:** 1 Department of Melanoma Medical Oncology, The University of Texas MD Anderson Cancer Center, 1515 Holcombe Blvd., Houston, TX 77030 USA; 2 Department of Genomic Medicine, The University of Texas MD Anderson Cancer Center, 1515 Holcombe Blvd., Houston, TX 77030 USA; 3 Department of Pathology, The University of Texas MD Anderson Cancer Center, 1515 Holcombe Blvd., Houston, TX 77030 USA; 4 Department of Sarcoma Medical Oncology, The University of Texas MD Anderson Cancer Center, 1515 Holcombe Blvd., Houston, TX 77030 USA; 5 Department of Surgical Oncology, The University of Texas MD Anderson Cancer Center, 1515 Holcombe Blvd, Houston, TX 77030 USA

**Keywords:** PRAME, Cancer testis antigen, Immunotherapy, Sarcoma, Sarcoma subtypes

## Abstract

**Background:**

PRAME (preferentially expressed antigen in melanoma), a member of the cancer-testis antigen family, has been shown to have increased expression in solid tumors, including sarcoma, and PRAME-specific therapies are currently in development for other cancers such as melanoma.

**Methods:**

To map the landscape of PRAME expression in sarcoma, we used publicly available data from The Cancer Genome Atlas (TCGA) and the Cancer Cell Line Encyclopedia (CCLE) projects and determined which sarcoma subtypes and subsets are associated with increased PRAME expression. We also analyzed how PRAME expression correlates with survival and expression of markers related to antigen presentation and T cell function. Furthermore, tumor and normal tissue expression comparisons were performed using data from the genotype-tissue expression (GTEx) project.

**Results:**

We found that uterine carcinosarcoma highly overexpresses the PRAME antigen, and synovial sarcomas and multifocal leiomyosarcomas also show high expressions suggesting that PRAME may be an effective target of immunotherapies of these tumors. However, we also discovered that PRAME expression negatively correlates with genes involved in antigen presentation, and in synovial sarcoma MHC class I antigen presentation deficiencies are also present, potentially limiting the efficacy of immunotherapies of this malignancy.

**Conclusions:**

We determined that uterine carcinosarcoma, synovial sarcoma, and leiomyosarcoma patients would potentially benefit from PRAME-specific immunotherapies. Tumor escape through loss of antigen presentation needs to be further studied.

## Background

Preferentially expressed antigen in melanoma (PRAME) was first discovered in melanomas and it was associated with cytotoxic T cell activation [1]. Shortly after its discovery, it was also shown to be expressed in acute leukemia cells [2]. The function of PRAME appears to be extensive though it was first identified as a repressor of the retinoic acid receptor pathway [3]. PRAME also inhibits myeloid differentiation in a retinoic acid-dependent and independent manner as well [4]. PRAME, like other cancer-testis antigens, has been shown be minimally expressed in adult human organs except for gonadal tissues and various human cancers including sarcomas.

Cancer-testis antigens such as MAGE-A and NY-ESO-1 have been widely explored, and these tumor-associated antigens have served as the therapeutic target of various vaccine strategies and adoptive cellular therapies. Objective tumor regressions of cutaneous metastases of melanoma patients have been documented with a MAGE-3.A1 peptide [5]. Similarly, patients with synovial sarcoma treated with genetically engineered autologous T cells with NY-ESO-1 recognition experienced RECIST partial responses as noted in 11 of 18 cases (61%) [6].

Various subtypes of sarcomas have demonstrated expression of cancer-testis antigens including synovial sarcomas, myxoid liposarcomas, chondrosarcomas, and osteosarcomas. Co-expression of PRAME and NY-ESO-1 has been shown to correlate with high-grade histologic features and a worse overall survival in patients with myxoid liposarcomas [7] and synovial sarcomas [8]. High protein expression levels of PRAME have been shown to correlate with a worse overall survival in osteosarcoma, and the expression of PRAME was more common in metastases compared to primary tumors [9]. In chondrosarcoma, a disease with low expression of PRAME at baseline, induction of PRAME with 5-aza-2-deoxycitabine rendered chondrosarcoma cells targetable by PRAME-specific CD8+ T cells [10].

The goal of this study is to evaluate the expression of PRAME across multiple sarcoma subtypes and normal tissues using three large public datasets. We report statistically significant associations to guide PRAME-specific therapies of sarcoma. To our knowledge this is the first comprehensive analysis of PRAME in multiple sarcoma subtypes and clinical subsets. In addition, we evaluated associations of T cell and antigen expression markers with PRAME expression to show how these may affect immunotherapies targeting this antigen.

## Methods

### Data sources

RNA expression and clinical data from the TCGA were downloaded from public repositories (https://tcga-data.nci.nih.gov). In the sarcoma TCGA, the following histologies were represented: leiomyosarcoma (LMS) (n = 106 samples), undifferentiated pleomorphic sarcoma/myxofibrosarcoma (UPS/MFS) (n = 76), dedifferentiated liposarcoma (DDLPS) (n = 58), synovial sarcoma (n = 10), and malignant peripheral nerve sheath tumors (MPNST) (n = 10). Data from carcinosarcoma cases (n = 57) were downloaded similarly from the uterine carcinosarcoma (UCS) TCGA project. Normal tissue expressions were obtained from the Genotype-Tissue Expression (GTEx, https://www.gtexportal.org/home/) project [11]. Homogeneous normal tissues were collapsed into a smaller number of groups the reduce figure complexity. Expression data of PRAME in cancer cell lines (n = 46) were downloaded from the website of the Cancer Cell Line Encyclopedia (CCLE) [12].

### Analysis of expression and clinical data

Clinical and mRNA expression data were merged into an input table using the TCGA sample identifiers. We included only those cases where both clinical and expression data were available. When comparing expressions from RNA sequencing from the TCGA and GTEx databases, we used the transcripts per million (TPM) unit, which was found to be most suitable unit for comparing RNA sequencing data [13]. For the analysis of PRAME expression in multifocal tumors, we used the *tumor_multifocal* TCGA clinical variable. Figures were created using the Tableau Desktop software. Kaplan–Meier analyses were performed using the ‘survival’ package of the R programming language. CCLE expression analyses were performed using microarray and also RNA-sequencing data. The TPM unit was used for RNA-seq, and in the case of microarray we used RMA-normalized data, which is calculated using a quantile normalization approach.

### Statistical analyses

For comparisons of two groups we performed two-tailed Student’s t-tests. When comparing multiple groups, we used Kruskal–Wallis rank sum tests followed by a posthoc Kruskal-Nemenyi test when p < 0.05. All differences were considered significant when p < 0.05, and a trend towards significance was noted when 0.05 ≤ p  <  0.1.

## Results

### PRAME is expressed in sarcoma and shows high overexpression in uterine carcinosarcoma

**Fig. 1:**
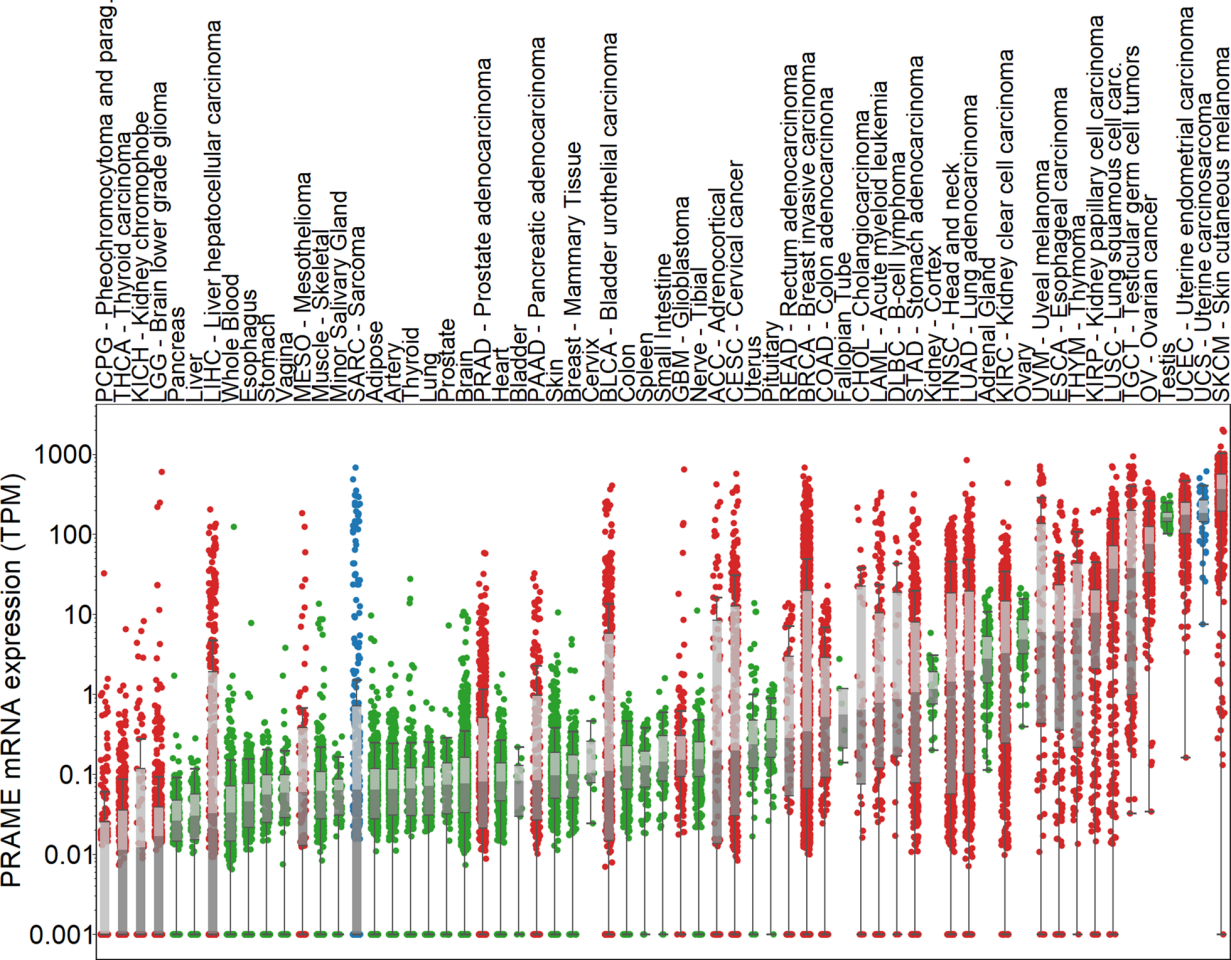
PRAME is overexpressed in sarcoma tumors. PRAME mRNA expression is displayed on the *y axis* for TCGA cancers and GTEx normal tissues, which are all shown in* columns*. Sarcoma samples in the sarcoma and uterine carcinosarcoma TCGAs are *blue*, while all other tumor samples are *red*. Normal tissue samples are *colored green*. Gene expressions equal to zero are shown at 0.001 TPM. *Boxes* around the median expression in tissue types represent quartiles. Tumor and normal tissues are sorted by median expression

To determine the relevance of PRAME as a target in sarcoma, we compared all normal (GTEx, n = 30 tissue types, n = 8153 samples) and tumor tissue (TCGA, n = 33 cancers) expressions (Fig. 1). Expression of PRAME in the uterine carcinosarcoma TCGA (n = 57) was significantly higher (p < 0.001) compared to the sarcoma TCGA as a whole, and only skin cutaneous melanoma showed a higher PRAME expression among all normal and tumor tissue types. Furthermore, PRAME expression was significantly higher (p < 0.001) in uterine carcinosarcoma than in normal uterus (n = 83).

### PRAME is expressed in sarcoma cell lines

**Fig. 2:**
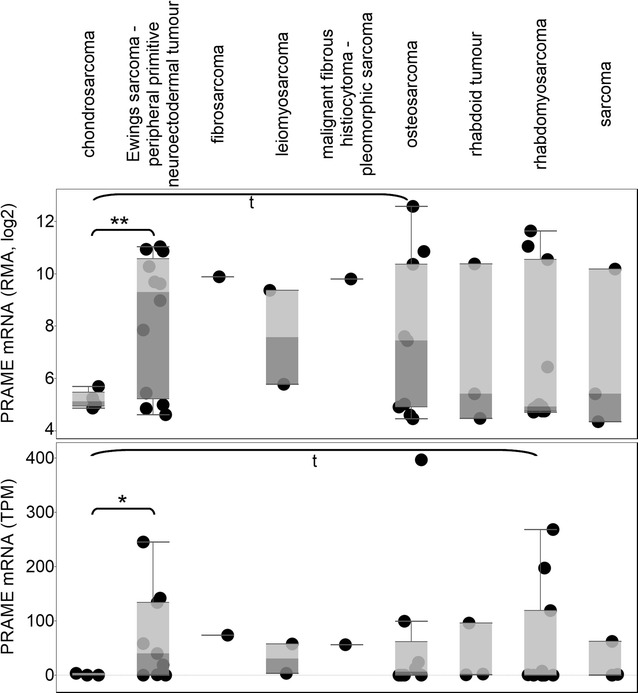
PRAME is expressed in sarcoma cell lines. Cancer Cell Line Encyclopedia sarcoma cell lines are shown in *columns*, each *dot* representing a cell line. PRAME expression is shown on the *y axis*. **p < 0.01, *p < 0.05, while t denotes a trend with 0.05 ≤ p < 0.1

In the CCLE cell line data (n = 46 with microarray, n = 40 with RNA-sequencing data) we found that sarcoma subtypes show diverse PRAME expressions (Fig. 2), however, in the microarray data, all four chondrosarcoma lines had lower expressions than other bone sarcoma cell lines such as Ewing’s sarcoma (p < 0.01) and osteosarcoma (p < 0.1). Analysis of the RNA-sequencing data confirmed chondrosarcoma—Ewing’s sarcoma difference (p < 0.05), and we also observed a trend for overexpression in rhabdomyosarcoma compared to chondrosarcoma (p < 0.1). Notably, with the exception of chondrosarcoma, PRAME over-expressing cell line(s) were found in all CCLE sarcoma types.

### PRAME is overexpressed in synovial sarcoma and in multifocal leiomyosarcoma

**Fig. 3:**
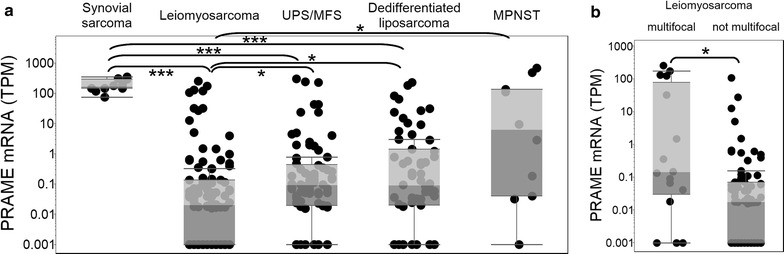
PRAME is overexpressed in synovial sarcoma and multifocal leiomyosarcoma. Expression of PRAME (*y axis*) is compared in subtypes of the sarcoma TCGA project (**a**), and in multifocal and non-multifocal leiomyosarcoma in the TCGA (**b**). Zero gene expression samples are shown at 0.001 TPM. *Grey boxes* around the median represent the two quartiles. Statistically significant differences between subtypes are denoted by *p < 0.05, and ***p < 0.001

Analyzing the expression of PRAME in sarcoma subtypes, we found that PRAME was highly expressed in all synovial sarcomas (Fig. 3a). The PRAME expression in these samples was significantly higher (p < 0.001) than in LMS, UPS/MFS, and DDLPS, while LMS expression was significantly lower (p < 0.05) than UPS/MFS, DDLPS, and MPNST PRAME expression (Fig. 3a). Importantly, a few of the LMS, UPS/MFS, DDLPS, and MPNST tumors also showed high PRAME expressions, suggesting that in addition to synovial sarcomas, these subtypes may also be considered for immunotherapies targeting PRAME. Although PRAME median expression was low in LMS, it showed a significantly higher (p < 0.05) expression in multifocal LMS compared to non-multifocal LMS (Fig. 3b), therefore PRAME may be a relevant target in multifocal LMS cases.

### PRAME expression negatively correlates with genes involved in antigen presentation

**Fig. 4:**
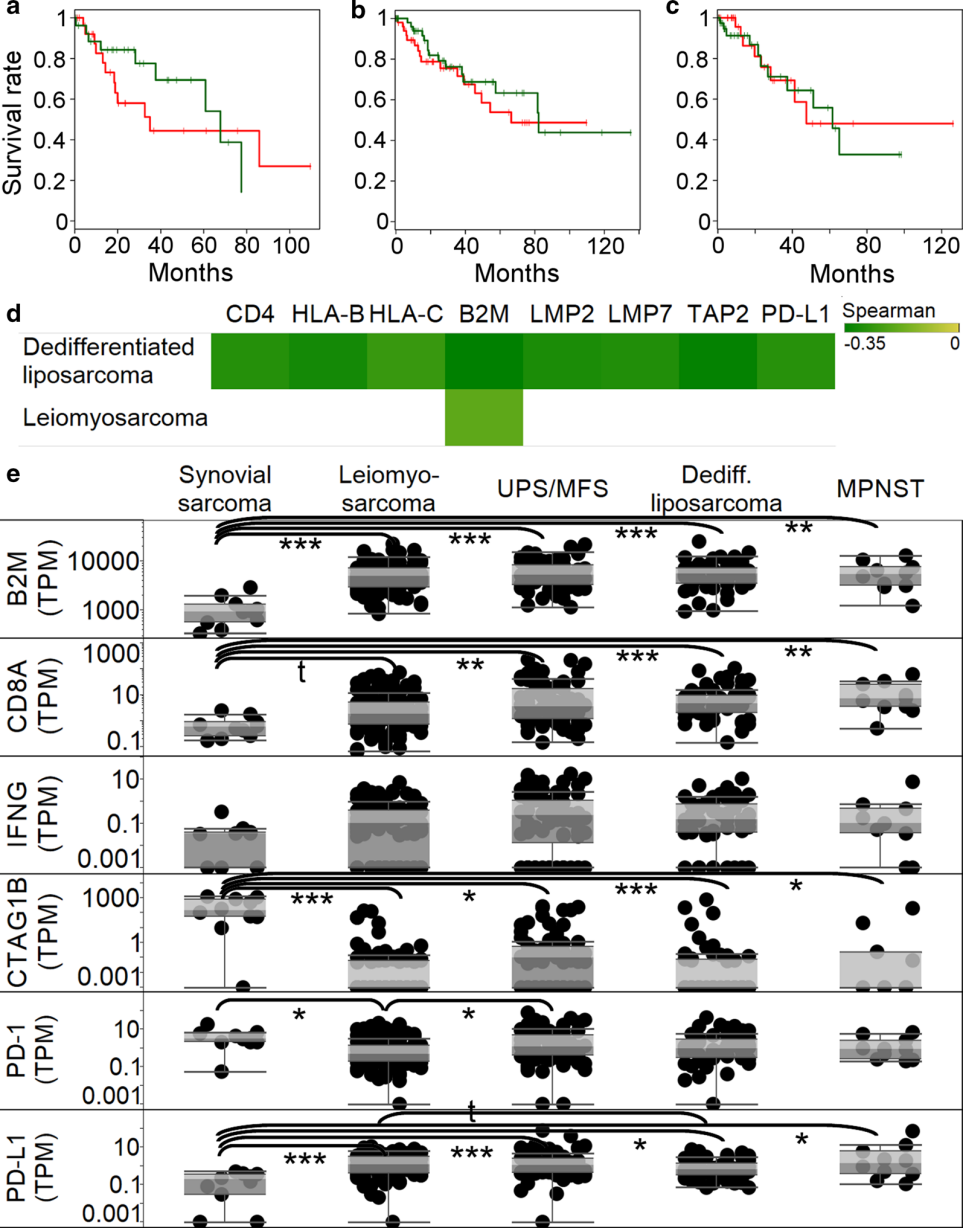
Survival and immune correlations of PRAME. Kaplan–Meier plots comparing low (*green*, below median expression samples) and high (*red*, above median expression) in dedifferentiated liposarcoma (**a**), leiomyosarcoma (**b**), and UPS/MFS (**c**) show no statistically significant associations. Spearman’s rank correlation coefficients of PRAME and antigen presentation and immune related genes are displayed in *panel* **d** (only p < 0.05 correlations are shown). Expression of B2M, CD8A, IFNG, CTAG1B (NY-ESO-1), PD-1, and PD-L1 are compared in sarcoma subtypes (**e**), where t: p < 0.1, *p < 0.05, **p < 0.01, and ***p < 0.001

We have determined that PRAME expression was not associated with overall survival in dedifferentiated liposarcoma (Fig. 4a), leiomyosarcoma (Fig. 4b), and UPS/MFS (Fig. 4c), subtypes where a sufficient number of samples were available for Kaplan–Meier analyses.

We also determined whether antigen presentation and other immune-related genes (B2M, CD3E, CD4, CD8A, GZMA, GZMB, HLA-A, HLA-B, HLA-C, IFNG, LCK, PRF1, LMP7, LMP2, TAP1, and TAP2) are associated with PRAME and found that expression of multiple genes involved in antigen presentation (HLA-B, HLA-C, B2M, LMP2, LMP7, TAP2) negatively correlate with PRAME expression in dedifferentiated liposarcoma and leiomyosarcoma (Fig. 4d). PD-L1 and PRAME expression also negatively correlated in dedifferentiated liposarcoma. The other three subtypes did not show significant (p < 0.05) correlations. Furthermore, we found that synovial sarcoma, which overexpresses PRAME, showed a significantly lower B2M and CD8A expressions compared to other subtypes (Fig. 4e). Interestingly, CTAG1B (NY-ESO-1) expression was significantly higher in synovial sarcoma than in the other sarcoma types (Fig. 4e). Furthermore, PD-1 expression was significantly higher (p < 0.05) in synovial sarcoma and UPS/MFS compared to leiomyosarcoma. Interestingly, PD-L1 was expressed in most subtypes except synovial sarcoma, where the expression level was significantly lower than in leiomyosarcoma (p < 0.001), UPS/MFS (p < 0.001), dedifferentiated liposarcoma (p < 0.05), and MPNST (p < 0.05).

## Discussion

Sarcomas represent a rare collection of neoplasms of mesenchymal origin that make up less than 1% of all cancer cases diagnosed each year in the United States [14]. While surgery can be curative for low-grade/low-stage disease, unresectable/metastatic disease is treated with systemic therapies. These therapies for soft tissue sarcomas have improved in slow incremental steps over the last 40 years with limited success [15]. Treatment of sarcoma is also difficult because more than one hundred subtypes have been identified. The success of immunotherapies in certain tumors provide a new avenue for investigation for the treatment of sarcoma. In fact, a recent phase II study (SARC028) of pembrolizumab for advanced/metastatic sarcomas demonstrated a response rate of 17% [16]. In the current study, we show that PRAME could be an effective immunotherapy target in specific sarcoma subtypes. Furthermore, we show that PD-1 and PD-L1 are also expressed in a heterogeneous manner, supporting further evaluation of anti-PD-1 and anti-PD-L1 therapies in sarcomas.

We determined that uterine carcinosarcoma, a disease that lacks standard therapies, highly overexpresses the PRAME antigen. Other cancer testis antigens have been identified as potential immunotherapy targets for this malignancy, including MAGE-A4 and NY-ESO-1 [17]. We propose that PRAME might also be an effective and broadly expressed target. Although the other sarcoma tumors included in the sarcoma TCGA showed a lower expression, a subset of samples was clearly characterized by PRAME overexpression. Multiple CCLE cell lines that were derived from sarcoma tumors retained PRAME expression, with the exception of chondrosarcoma, which was not included in the sarcoma TCGA.

Our analysis of PRAME expression associations with subtypes and clinical variables revealed that PRAME is overexpressed in synovial sarcoma and in multifocal leiomyosarcoma. Notably, all other sarcoma subtypes showed a highly heterogeneous PRAME expression, from zero to very high expression. This is in line with heterogeneity of sarcomas, which represents a major challenge. Cancer testis antigens, focusing primarily on NY-ESO-1, are currently being tested in clinical trials and show promise as targets of adoptive immunotherapies and cancer vaccines to treat sarcoma [18]. PRAME co-overexpression with NY-ESO-1 in synovial sarcoma suggests that this antigen may also be targeted efficiently by these immunotherapy approaches.

The negative correlation that we identified between PRAME and expression of antigen presentation-related genes also supports that tumor-associated antigens derived from the PRAME protein can be recognized by the immune system, and tumors lose expression of genes involved in antigen presentation to avoid an effective immune response. Indeed, in the PRAME-overexpressing synovial sarcoma, beta2-microglobulin (B2M) loss appears to be a mechanism by which the tumor avoids immune recognition, evidenced by low expression of the CD8A cytotoxic T cell marker. However, we also found non-zero interferon gamma (IFNG) expression in half of the synovial sarcoma samples that we analyzed, therefore we hypothesize that there are functional and active T cells in those tumors that may be exploited to develop effective immunotherapies. Furthermore, MHC class I recovery approaches [19] might also be needed to ensure the success of T cell-based immunotherapy of sarcoma.

## Conclusions

Our analysis of sarcoma subtypes shows that uterine carcinosarcoma, synovial sarcoma, and multifocal leiomyosarcoma samples overexpress this antigen and patients with these malignancies would potentially benefit from PRAME-specific immunotherapies. We also found a negative correlation between PRAME and expression of genes involved in antigen presentation, which may provide a way for tumors to avoid immune recognition.

## Abbreviations

LMS: leiomyosarcoma
UPS/MFS: undifferentiated pleomorphic sarcoma/myxofibrosarcoma
DDLPS: dedifferentiated liposarcoma
MPNST: malignant peripheral nerve sheath tumors
TCGA: The Cancer Genome Atlas
GTEx: genotype-tissue expression project
TPM: transcripts per million
